# Differential responses of rabbit ventricular and atrial transient outward current (I_to_) to the I_to_ modulator NS5806

**DOI:** 10.14814/phy2.13172

**Published:** 2017-03-07

**Authors:** Hongwei Cheng, Mark B. Cannell, Jules C. Hancox

**Affiliations:** ^1^ School of PhysiologyPharmacology and NeuroscienceBiomedical Sciences BuildingUniversity WalkBristolU.K

**Keywords:** action potential, flecainide, I_to_, Kv4.3, Kv 4.2, Kv1.4, NS5806, potassium channels, transient outward current

## Abstract

Transient outward potassium current (I_to_) in the heart underlies phase 1 repolarization of cardiac action potentials and thereby affects excitation–contraction coupling. Small molecule activators of I_to_ may therefore offer novel treatments for cardiac dysfunction, including heart failure and atrial fibrillation. NS5806 has been identified as a prototypic activator of canine I_to_. This study investigated, for the first time, actions of NS5806 on rabbit atrial and ventricular I_to_. Whole cell patch‐clamp recordings of I_to_ and action potentials were made at physiological temperature from rabbit ventricular and atrial myocytes. 10 *μ*mol/L NS5806 increased ventricular I_to_ with a leftward shift in I_to_ activation and accelerated restitution. At higher concentrations, stimulation of I_to_ was followed by inhibition. The EC_50_ for stimulation was 1.6 *μ*mol/L and inhibition had an IC_50_ of 40.7 *μ*mol/L. NS5806 only inhibited atrial I_to_ (IC_50_ of 18 *μ*mol/L) and produced a modest leftward shifts in I_to_ activation and inactivation, without an effect on restitution. 10 *μ*mol/L NS5806 shortened ventricular action potential duration (APD) at APD_20_‐APD_90_ but prolonged atrial APD. NS5806 also reduced atrial AP upstroke and amplitude, consistent with an additional atrio‐selective effect on Na^+^ channels. In contrast to NS5806, flecainide, which discriminates between Kv1.4 and 4.x channels, produced similar levels of inhibition of ventricular and atrial I_to_. NS5806 discriminates between rabbit ventricular and atrial I_to,_ with mixed activator and inhibitor actions on the former and inhibitor actions against the later. NS5806 may be of significant value for pharmacological interrogation of regional differences in native cardiac I_to_.

## Introduction

Genetically distinct potassium (K^+^) ion channel currents are responsible for the repolarization of cardiac action potentials (APs) (Tamargo et al. 2004). The rapid and slow delayed rectifier K^+^ currents (I_Kr_ and I_Ks_) contribute to ventricular AP repolarization over plateau voltages, while the inward rectifier K^+^ current (I_K1_) plays key roles in both terminal repolarization and in setting the resting membrane potential of nonpacemaker myocytes (Nerbonne 2000; Tamargo et al. 2004). In many species, including (for example) man, dog, ferret, rabbit, and rodent, initial rapid repolarization (phase 1) takes place before the AP plateau (phase 2). This arises from a combination of rapid inactivation of fast Na^+^ current (I_Na_) and from the activation of a voltage‐dependent transient outward K^+^ current (I_to_) and, in the atria, of ultrarapid delayed rectifier K^+^ current (I_Kur_) (Nerbonne 2000; Tamargo et al. 2004). The pore‐forming subunits of channels that underlie I_to_ are derived from *KCND3* (Kv4.3), *KCND2* (Kv4.2), and *KCNA4* (Kv1.4) genes (Nerbonne and Kass 2005; Niwa and Nerbonne 2010). Kv4.2 and 4.3 are believed to underlie an I_to_ that exhibits fast recovery kinetics (I_to,f_), whilst Kv1.4 is responsible for I_to_ with slower kinetics (I_to,s_) (Nerbonne and Kass 2005; Niwa and Nerbonne 2010). Regional and species differences in I_to_ are likely to result from the relative balance between these I_to_ subtypes (Niwa and Nerbonne 2010). Native I_to,f_ channels require interactions between Kv4.x and K^+^ Channel interacting Protein 2 (KChIP2), while other proteins (Kv*β*, DPP6 and members of the KCNE family) may also modulate the current (Radicke et al. 2006; Niwa and Nerbonne 2010).

I_to_ contributes to phase 1 repolarization, but can also affect both the plateau (phase 2) and repolarization (phase 3) of the AP, due to the time‐ and voltage‐dependent behavior of I_Kr_, I_Ks_, and L‐type Ca^2+^ current (I_Ca,L_) (Nerbonne 2000; Niwa and Nerbonne 2010). Reductions in I_to_ are seen in heart failure (HF) and human atrial fibrillation, and abnormal I_to_ regulation may also contribute to Brugada syndrome (Brandt et al. 2000; Antzelevitch 2006; Niwa and Nerbonne 2010). Indeed, pharmacological modification of I_to_ coupled with I_Ca,L_ block has recently been utilized as a way of studying electrogram fractionation in Brugada syndrome (Szel and Antzelevitch 2014; Patocskai et al. 2016). On the other hand, action potential clamping has shown that a loss of I_to_ in human APs directly leads to reduced and dyssynchronous Ca^2+^ release, raising the possibility that pharmacological I_to_ activation may have therapeutic value in HF (Cooper et al. 2010) (see also (Sah et al. 2003)). Consistent with this idea, data obtained from a canine HF model, using a single NS5806 concentration of 10 *μ*mol/L to stimulate I_to_, suggest that ventricular I_to_ stimulation may be able to mitigate electrophysiological changes in HF (Cordeiro et al. 2012).

In experiments on recombinant Kv4.x and Kv1.4 channels, the response of Kv4.3 to NS5806 has been shown to be modulated by co‐expression with KChIP2, while current carried by recombinant Kv1.4 channels was inhibited rather than activated by the compound (Lundby et al. 2010). It follows that the net effect of NS5806 on native I_to_ may vary both with the levels of Kv4.x/1.4 isoforms expressed as well as their possible association with KChIP2. To our knowledge, all studies to date of NS5806 effects on native I_to_ have used the dog (Calloe et al. 2009, 2010, 2011; Cordeiro et al. 2012) and the effect of NS5806 on native human ventricular I_to_ has yet to be ascertained. Some differences between canine and human I_to_ have been reported (Akar et al. 2004; Jost et al. 2013). Rabbits are widely used in studies of cardiac electrophysiology and can provide a cost‐effective alternative to larger species such as dog, while possessing ventricular action potentials closer to human than those from rats or mice (Milani‐Nejad and Janssen 2014; Camacho et al. 2016). Normal rabbit atrial and ventricular tissue each express Kv1.4, 4.2 and 4.3 (Wang et al. 1999; Bosch et al. 2003; Rose et al. 2005), and Kv1.4, Kv4.2, and Kv4.3 have all been detected by RT PCR in human ventricle (Kaab et al. 1998; Gaborit et al. 2007) although only the presence of Kv1.4 and Kv4.3 have been confirmed by Western blotting (Akar et al. 2004). While rabbit I_to_ is known to be slower than human I_to_ to recover from inactivation (e.g., Fermini et al. 1992; Mitcheson and Hancox 1999), it is nevertheless instructive to determine the effects of NS5806 on I_to_ from this species both to further knowledge of modulation of I_to_ from a widely used model species and for comparison with available information on canine I_to_. The aim of this paper, therefore, was to study the modulatory effects of NS5806 on rabbit ventricular and atrial I_to_. The results reveal distinct responses of rabbit atrial and ventricular I_to_ to NS5806.

## Materials and Methods

### Rabbit ventricular and atrial myocyte isolation and storage

Myocytes were isolated from the right ventricle and left atrium of hearts of male New Zealand White rabbits (2–3 kg). All procedures were in accordance with the UK Home Office Animals (Scientific Procedures) Act, 1986 and had institutional ethical approval. Ventricular and atrial myocytes were isolated by enzymatic and mechanical dispersion, using previously described methods (Hancox et al. 1993; Howarth et al. 1996). Cells were temporarily stored in a Kraft‐Brühe solution (Isenberg and Klockner 1982) at 4°C prior to electrophysiological recording.

### Electrophysiological recording

Myocytes were placed in an experimental chamber mounted on an inverted microscope (Nikon Eclipse TE2000‐U) and superfused with a standard ‘normal’ Tyrode's solution containing (in mmol/L): 140 NaCl, 4 KCl, 2 CaCl_2_, 1 MgCl_2_, 10 glucose, 5 HEPES (pH 7.4 with NaOH). This solution was used in all experiments to obtain the whole‐cell recording mode and was also used as superfusate for action potential measurements. For I_to_ measurements, the above solution was modified as previously described (Mitcheson and Hancox 1999): N‐methyl‐D‐glucamine (NMDG) chloride was substituted for NaCl and 20 *μ*mol/L nifedipine was used to inhibit I_Ca,L_. During experimental recordings, the superfusates were applied to the cell, using a home‐built device capable of exchanging solution bathing the cell in <1 sec (Levi et al. 1996). Borosilicate patch pipettes (A‐M Systems Inc, Sequim, WA) were pulled, using a Narishige vertical puller and fire‐polished (PP‐830 and MF83, Narishige Japan) to a resistance of 2–3 MΩ. For I_to_ recording, pipettes were filled with a solution containing (in mmol/L): 113 KCl, 10 HEPES, 0.4 MgCl_2_, 5 glucose, 5 K_2_ATP, 5 K_4_BAPTA (pH 7.2 with KOH). For AP recording, the pipette solution contained (in mmol/L): 110 KCl, 10 NaCl, 0.4 MgCl_2_, 10 HEPES, 5 glucose, 5 K_2_ATP, 0.5 GTP‐Tris (pH 7.1 with KOH). Series resistance values (typically 4–7 MΩ) were compensated by >70%. All recordings were made at 35–37°C. NS5806 (1‐[3,5‐bis(trifluoromethyl)phenyl]‐3‐[2,4‐dibromo‐6‐(2H‐tetrazol‐5‐yl)phenyl]urea) was obtained from Tocris (Bristol, UK) and dissolved in DMSO to produce stock solutions between 1 and 100 mmol/L (stored at −20°C). Stock solutions were diluted with the external solutions to obtain the final concentrations as given in the Results, with a final DMSO concentration in the superfusate of 1 in 1000 vv. Higher concentrations of stock solution in DMSO showed poor solubility in our hands, limiting the maximum concentration tested in the experimental solutions to 100 *μ*mol/L. Flecainide was obtained from Sigma‐Aldrich (UK), and dissolved in distilled water to produce stock solutions between 1 and 100 mmol/L.

### Data analysis

Data are presented as mean ± SEM, except for EC_50_/IC_50_ values derived from concentration‐response plots, for which 95% confidence intervals are given. Statistical analyses were performed, using Microsoft Excel (Microsoft) and Prism (GraphPad Software Inc.) and fits to particular datasets were made using either Prism or the Clampfit module of pClamp 10 (Axon Instruments, Molecular Devices). Statistical comparisons employed paired or unpaired *t‐*test, 1 or 2‐way ANOVA (with Bonferroni post‐test) as appropriate (*P *<* *0.05 was taken as statistically significant).

## Results

### Concentration‐dependent effects of NS5806 on ventricular and atrial I_to_

**Figure 1:**
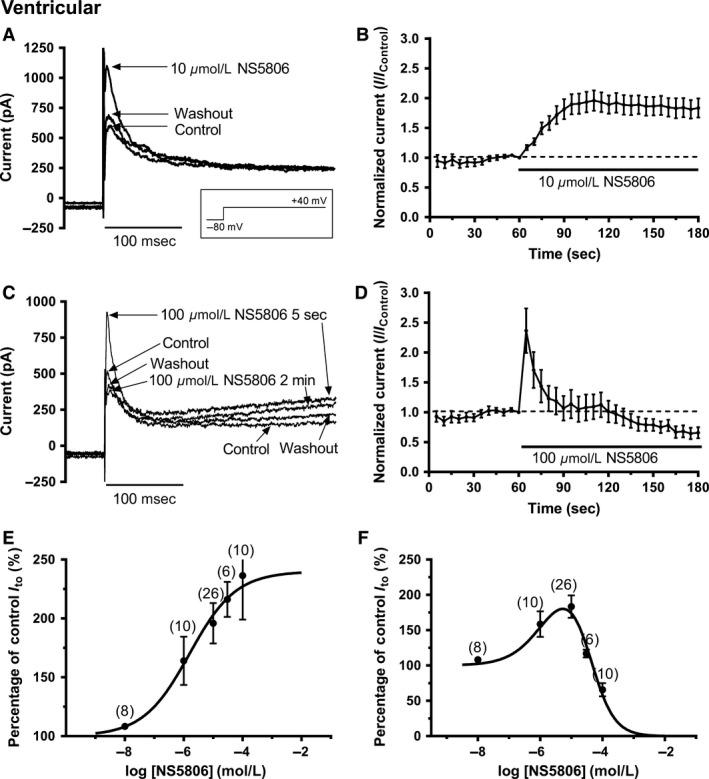
*NS5806 modulation of I*_*to*_ *from rabbit ventricular cells*. (A) Representative current records in control, after application of 10 *μ*mol/L NS5806 and after washout with control solution. The voltage protocol is shown as an inset. (B) Mean (± SEM,* n* = 26) time course of response to 10 *μ*mol/L NS5806 of I_to_ (measured as peak minus end‐pulse current), using protocol shown in A. (C) Representative current records in control, after application of 100 *μ*mol/L NS5806 and after washout with control solution. The voltage protocol is the same as that used for panel A. (D) Mean (± SEM,* n* = 10) time course of response of I_to_ (peak minus end‐pulse current) to 100 *μ*mol/L NS5806, using protocol shown in A. Note the initial stimulation followed by inhibition. (E) Concentration‐dependence of the maximal agonist effect of NS5806 on I_to_. Data (for peak minus end‐pulse current effects) were fitted with a standard Hill‐equation to get the EC_50_ and n_H_ values given in the ‘Results’ text. Values in parentheses denote number of independent replicates at each concentration. (F) Concentration–response relation for the steady‐state effect of NS5806 on I_to_. For each cell, the effect of NS5806 at 120 sec was recorded and current amplitude expressed as a % of control. Data (for peak minus end‐pulse current effects) were fitted with a two site (agonist and antagonist) Hill‐equation to get the EC_50_/IC_50_ and n_H_ values. Values in parentheses denote number of independent replicates at each concentration.

Prior canine studies have employed a single NS5806 concentration of 10 *μ*mol/L for I_to_ experiments. Here, a wide range of concentrations (10 nmol/L to 100 *μ*mol/L) was investigated against ventricular I_to_. An exemplar ventricular I_to_ activated by depolarization from −80 mV to +40 mV in control solution, in the presence of 10 *μ*mol/L NS5806 and following washout is shown in Figure 1A. The marked augmentation of I_to_ amplitude by NS5806 is apparent; this effect was largely reversible on drug washout. Current remaining after the initial time‐dependent, inactivating component was not altered by NS5806 at this concentration. Figure 1B shows the mean time course for augmentation of time‐dependent (peak minus end‐pulse) ventricular I_to_ at +40 mV by 10 *μ*mol/L NS5806 (*n* = 26): the maximal response was seen within 1 min of drug application. The increase in I_to_ amplitude was accompanied by acceleration of I_to_ inactivation time course (mean inactivation t_half_ = 30.3 ± 2.4 msec in control and 21.5 ± 1.2 msec in 10 *μ*mol/L NS5806; *P* < 0.01, *n* = 26). Despite this modest acceleration, the integral of the inactivating current was increased to 150.7 ± 10.5% of control (*P* < 0.01). Four additional concentrations of NS5806 were tested. At 1 *μ*mol/L and 10 nmol/L, qualitatively similar but smaller responses to that with 10 *μ*mol/L were seen. However, at higher concentrations (30 and 100 *μ*mol/L), the response of peak I_to_ to NS5806 became biphasic with an initial increase in peak I_to_ followed by a decrease. Figure 1C shows representative traces for the effects of 100 *μ*mol/L NS5806. The initial peak I_to_ (trace at 5 sec) showed a rapid increase in amplitude compared to control, but then declined to a level below that in control solution (trace at 2 min); this effect was poorly reversible. An additional effect of this concentration was a progressive increase in outward current following the initially inactivating current component. This secondary effect was partially reversible on washout. Figure 1D shows the time course of the biphasic effect of 100 *μ*mol/L NS5806 on peak minus end‐pulse current amplitude (*n* = 10). In order to quantify the concentration‐dependence of NS5806 action, two concentration‐response relations were constructed: Figure 1E shows the relationship for the maximal stimulatory effect of the compound, whilst Figure 1F shows the relationship at steady‐state effect. The derived EC_50_ for augmentation of peak minus end‐pulse I_to_ (Fig. 1E) was 1.6 *μ*mol/L (LogEC_50_ mean ± SEM: −5.80 ± 0.12; 95% C.I: 0.6–3.9 *μ*mol/L), with a Hill slope of 0.55 ± 0.08. For a similar plot for augmentation of the peak current amplitude (not shown), the derived EC_50_ was also 1.6 *μ*mol/L (LogEC50 mean±SEM: −5.81 ± 0.21; 95% C.I: 0.3–7.1 *μ*mol/L), with a Hill slope of 0.58 ± 0.14. The peak minus end‐pulse data in Figure 1F could not be described by a single Hill equation, but could be fitted by two site model in which the EC_50_ describing augmentation of I_to_ was fixed to the value obtained from Figure 1E (1.6 *μ*mol/L), whilst the IC_50_ value derived for the descending phase of the relationship was 40.7 *μ*mol/L (LogIC_50_ mean ± SEM: −4.39 ± 0.13; 95% C.I: 11.7–112.2 *μ*mol/L), with a Hill slope of −1.15 ± 0.22. A similar analysis of the biphasic effect of NS5806 on the peak current amplitude (not shown), again utilizing an EC_50_ of 1.6 *μ*mol/L for augmentation of I_to_, yielded an IC_50_ for the descending phase of the relationship of 21.2 *μ*mol/L (LogIC_50_ mean ± SEM: −4.67 ± 0.30; 95% C.I: 10 *μ*mol/L to 143 mmol/L), with minimum of 74% of control and a Hill slope of −1.09 ± 0.50.

**Figure 2:**
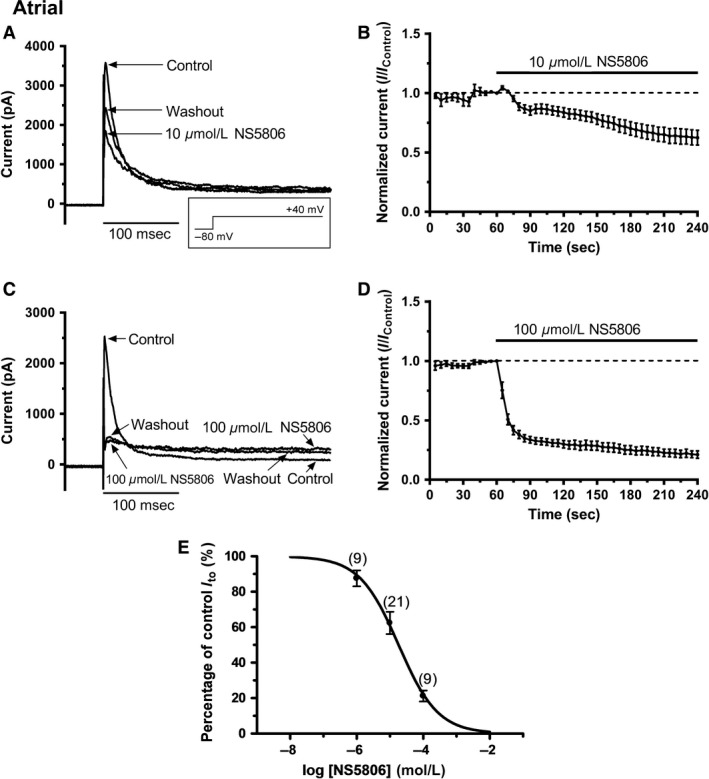
*NS5806 inhibition of I*_*to*_ *from rabbit atrial cells*. A: Representative current records in control, 10 *μ*mol/L NS5806 and washout with control solution elicited by the protocol shown as an inset (same protocol as Figure 1). B: Mean (± SEM,* n* = 21) time course of response to 10 *μ*mol/L NS5806 of I_to_ (measured as peak minus end‐pulse current, using protocol shown in A. C: Representative current records in control, after application of 100 *μ*mol/L NS5806 and after washout with control solution. The voltage protocol is the same as that used for panel A. D: Mean (± SEM,* n* = 9) time course of response of I_to_ (peak minus end‐pulse current) to 100 *μ*mol/L NS5806, using protocol shown in A. E: Isochronal concentration–response relation for the inhibition of atrial I_to_ by NS5806. For each cell, the effect of NS5806 at 180 s (on peak minus end‐pulse currents) was recorded and current amplitude expressed as a % of control. Data were fitted with a Hill‐equation to get the IC_50_ and n_H_ values given in the ‘Results’ text.

Figure 2A shows representative traces of atrial I_to_ activated by depolarization from ‐80 mV to +40 mV in control solution, in the presence of 10 *μ*mol/L NS5806 and following washout. In contrast to the effects seen on ventricular I_to_, NS5806 *reduced* atrial I_to_ amplitude and this was accompanied by a modest slowing of I_to_ inactivation time course (inactivation t_half_ in control of 13.5 ± 0.9 msec and in 10 *μ*mol/L NS5806 of 17.3 ± 1.0 msec; *P* < 0.01, *n* = 21). The current remaining after the initial time‐dependent inactivating current was little affected by this concentration of NS5806. The integral of inactivating current in 10 *μ*mol/L NS5806 for atrial cells decreased to 70.9 ± 6.6% of control (*P* < 0.01) and inhibitory effects of this NS5806 concentration did not fully reverse on washout. Figure 2B shows the mean time course of action of 10 *μ*mol/L NS5806 (*n* = 21) on peak minus end‐pulse I_to_. Figure 2C contains representative traces showing the effect of 100 *μ*mol/L NS5806. The rapidly activating peak I_to_ was strongly suppressed at this concentration of NS5806. Residual current was somewhat elevated but no progressively activating outward current was seen at this concentration, in contrast to the effect seen in ventricular cells (compare Fig. 1C and 2C). Figure 2D shows the mean time course of action of 100 *μ*mol/L NS5806 (*n* = 9) on peak minus end‐pulse I_to_. Figure 2E shows mean concentration‐response data for 1, 10 and 100 *μ*mol/L on peak minus end‐pulse current. A fit to these data with a one site Hill equation yielded an IC_50_ of 18.2 *μ*mol/L (LogIC_50_ mean ± SEM: −4.74 ± 0.05; 95% C.I: 4.2–80.0 *μ*mol/L; Hill coefficient: −0.74 ± 0.06). Analysis of peak current inhibition gave an IC_50_ of 34.7 *μ*mol/L (LogIC50 mean ± SEM: −4.46 ± 0.04; 95% C.I: 11.9–101.8 *μ*mol/L; Hill coefficient: −0.54 ± 0.03).

### Effects of NS5809 on voltage‐dependent activation and inactivation of I_to_

**Figure 3:**
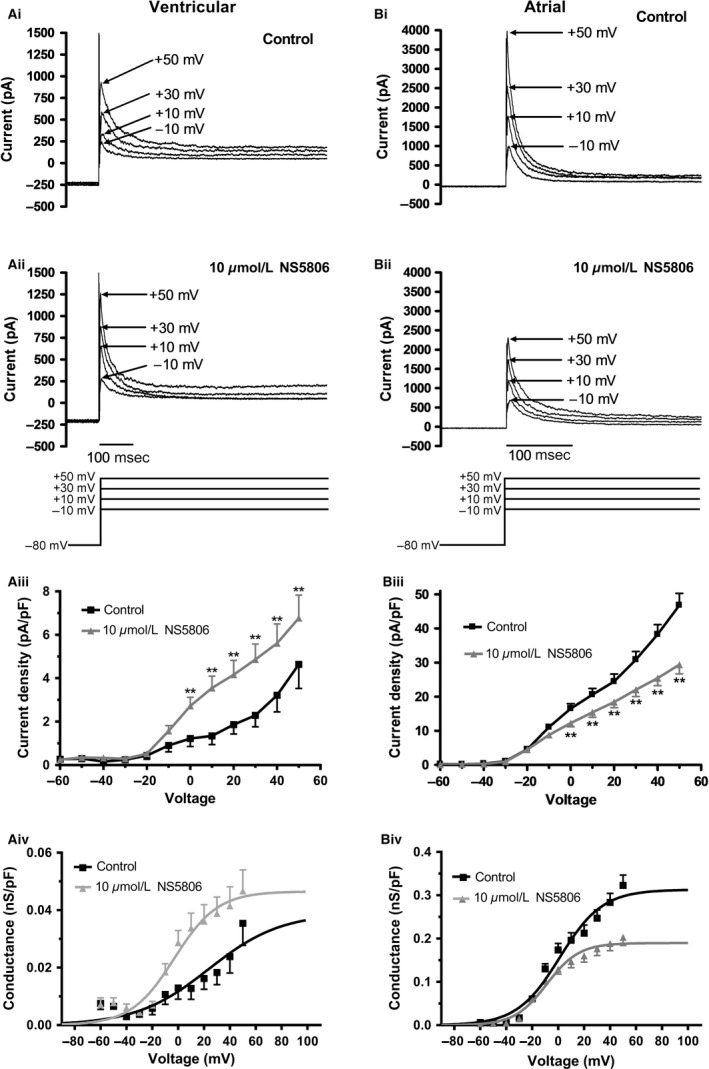
*Effects of NS5806 on voltage dependence of I*_*to*_ *activation*. Ai‐Aii: Representative ventricular current traces with control solution (Ai) and 10 *μ*mol/L NS5806 (Aii) at the potentials indicated (protocol shown as lower panel of Aii). From the holding potential of ‐80 mV, an initial 1‐second duration ‘conditioning’ step was applied to potentials between −90 and +50 mV in 10 mV increments. The conditioning step both enabled activation of I_to_ (on depolarization) and also enabled subsequent inactivation during the maintained depolarization. A second 500 msec ‘test’ step to +40 mV was applied to determine how availability (inactivation) of I_to_ was influenced by the conditioning pulse. A brief (3‐msec) step at −80 mV was included between the first and second steps to ensure that any residual capacitance artefacts that occurred during the test depolarization were not influenced by differing conditioning voltages. Interpulse interval was 5 sec. Aiii: Mean I‐V relations (normalized to cell capacitance) for ventricular I_to_ elicited by the initial 1s step of protocol described above, in control and in 10 *μ*mol/L NS5806 (same protocol as Ai,Aii; *n* = 7). Control data are shown in black; NS5806 data are shown in gray (error bars indicate SEM). ** denotes significant difference at *P* < 0.01. Aiv: Voltage‐dependence of conductance for ventricular I_to_ (same experiments as shown in Aiii). Data were fitted with a Boltzmann equation of the form: G/G_max_=1/[1 + exp[(V_0.5_‐V)/k_a_]], where G=conductance at test voltage V, G_max_= maximal conductance, V_0.5_=half‐maximal activation voltage, and k_a_=activation slope factor. V_0.5_ and k_a_ values are given in the ‘Results’ text. Bi‐Bii: Representative atrial current traces with control solution (Bi) and 10 *μ*mol/L NS5806 (Bii) at the potentials indicated. The protocol was the same as for ventricular cells as shown in Ai. Biii: Mean I‐V relations (normalized to cell capacitance) for atrial I_to_ in control and in the presence of 10 *μ*mol/L NS5806 (same protocol as Bi,Bii; *n* = 8). Control data are shown in black (and with +SEM bars); NS5806 data are shown in gray (and with ‐SEM bars). ** denotes significant difference between control and NS5806 at *P* < 0.01. Biv: Voltage‐dependent activation curves for atrial I_to_ (data from same experiments as Biii). For each experiment and each recording condition (control and NS5806) macroscopic conductance values were calculated at each voltage, normalized to maximal conductance during the protocol and pooled data fitted with the Boltzmann equation as described above.

**Figure 4:**
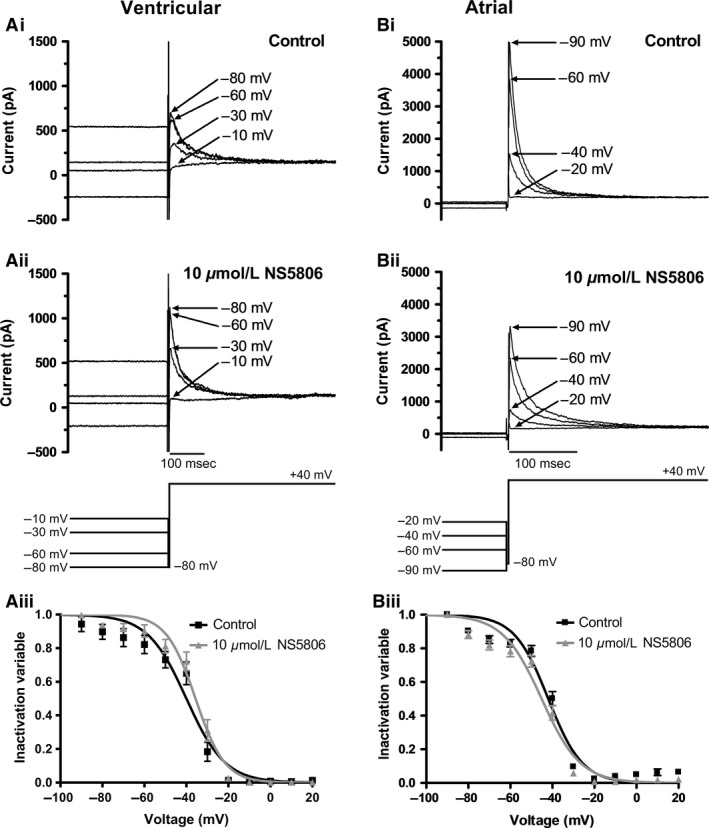
*Effect of NS5806 on voltage‐dependent inactivation of I*_*to*_. Ai–Aii: Representative ventricular current traces with control solution (Ai) and 10 *μ*mol/L NS5806 (Aii) elicited by protocol shown as lower panel of Aii. Full protocol contained 1 sec conditioning steps in 10 mV increments between −90 mV and +20 mV, followed by a 500 msec test pulse to +40 mV. Conditioning and test steps were separated by a brief (3 msec) period at −80 mV. The figure focuses on currents elicited by the test step following conditioning steps to the voltages indicated. Currents at selected voltages are shown for clarity of display. Aiii: Mean (± SEM) plots of inactivation variables against conditioning voltage in control and in the presence of 10 *μ*mol/L NS5806 (*n* = 7). For each experiment and each condition, currents during each test command were normalized to the maximal test current observed during the protocol, pooled and plotted against conditioning voltage. Data were fitted by a Boltzmann function: I/I_max_=1‐[1/[1 + exp((V_0.5_‐V)/k_i_)]], where I=current during the test pulse (+40 mV), V= conditioning voltage, I_max_= maximal test current, V_0.5_=half‐maximal inactivation voltage, and k_i_=inactivation slope factor. V_0.5_ and k_i_ values are given in the Results text. Bi–Bii: Representative atrial current traces with control solution (Bi) and 10 *μ*mol/L NS5806 (Bii) elicited by protocol shown as lower panel of Bii. Voltage protocol as described for ‘A’. The figure shows currents elicited by the test step after selected conditioning steps (voltages indicated on traces). Biii: Mean (± SEM) plots of atrial inactivation variables against conditioning voltage in control and NS5806 (*n* = 8). Data were fitted by the Boltzmann equation described in ‘A’. V_0.5_ and *k*_*i*_ values are given in the Results text.

The voltage dependence of activation and inactivation of I_to_ were determined, using a classical Hodgkin‐Huxley protocol ((Mitcheson and Hancox 1999); see Figure 3 and Figure 4 legends for details). Figures 3Ai and Aii show families of ventricular I_to_ elicited by depolarization to a range of membrane potentials both in the absence and presence of 10 *μ*mol/L NS5806. Peak I_to_ was increased by 10 *μ*mol/L NS5806 (*n* = 7) at all potentials greater than 0 mV, as shown the current‐voltage (I‐V) plots in Figure 3Aiii (data normalized to cell capacitance). No significant difference in mean end‐pulse current was seen between control and 10 *μ*mol/L NS5806 between −60 and +50 mV (*P* > 0.05). Figure 3Aiv shows the voltage dependence of I_to_ activation derived from normalized conductance voltage (G‐V) plots, with Boltzmann fits used to derive half‐maximal activation voltage (V_0.5_) and slope factor (*k*_*a*_). In control solution, ventricular I_to_ activation V_0.5_ was +25.3 ± 2.6 mV (*k*_*a*_=25.4 ± 3.1 mV), whilst in 10 *μ*mol/L NS5806 V_0.5_ was −3.4 ± 2.7 mV (*P* < 0.01 versus control; *k*_*a*_ = 16.7 ± 1.1 mV, also *P* < 0.01 versus control).

Figures 3Bi and Bii show families of atrial I_to_ during depolarizations to a range of voltages and demonstrate that, in marked contrast to ventricular myocytes, peak I_to_ was decreased by 10 *μ*mol/L NS5806 over the range of potentials tested. Mean I‐V relations in control and after application of 10 *μ*mol/L NS5806 (*n* = 8; normalized to cell capacitance) are shown in Figure 3Biii and NS5806 significantly reduced I_to_ amplitude at all voltages greater than 0 mV. No significant difference in mean end‐pulse current was seen between control and 10 *μ*mol/L NS5806 between −60 and +50 mV (*P* > 0.05). Figure 3Biv shows normalized G‐V plots of atrial I_to_ fitted with a Boltzmann function to derive activation parameters. The activation V_0.5_ for atrial I_to_ in control was 2.8 ± 2.5 mV (*k*_*a*_=16.7 ± 1.3 mV), whilst in NS5806 it was ‐8.6 ± 1.5 mV (*P* < 0.01 versus control; *k*_*a*_ = 12.3 ± 0.9 mV, also *P* < 0.01 vs. control).

Thus, NS5806 produced a leftward shift and decrease in slope in the voltage dependence of activation of I_to_ in both cell types, though the magnitude of this effect was much greater in ventricular than atrial myocytes.

Figures 4Ai and Aii show families of I_to_ elicited by the test depolarization in ventricular myocytes following different conditioning steps in both control (Fig. 4Ai) and after adding 10 *μ*mol/L NS5806 (Fig. 4Aii). Under both conditions, I_to_ was greater at more negative conditioning voltages. After normalizing the test pulse I_to_ to the maximal test I_to_ observed following the different conditioning pulses protocol and fitting a Boltzmann function (Fig. 4Aiii; *n* = 7) the half‐maximal inactivation voltage (V_0.5_) and slope factor (*k*_*i*_) values were not significantly changed by NS5806 (Control: V_0.5_ of ‐40.9 ± 2.7 mV; *k*_*i*_ *=* 9.1 ± 1.7 mV and with NS5806: V_0.5_ of −36.2 ± 2.0 mV; *k*_*i*_ = 6.9 ± 1.1 mV; *P* > 0.1 for both).

Figure 4Bi and Bii shows equivalent data for atrial I_to_ in control and NS5806 and Figure 4Biii shows mean data and Boltzmann fits. In eight experiments the mean atrial I_to_ inactivation V_0.5_ in control was ‐42.3 ± 1.4 mV which was shifted to −45.6 ± 1.6 mV in 10 *μ*mol/L NS5806 (*P* < 0.01). The slope factors appeared unchanged; in control *k*_*i*_ was 8.0 ± 1.0 mV and in NS5806 *k*_*i*_ was 9.1 ± 1.1 mV (*P* > 0.05 vs. control). Thus, NS5806 produced a modest but significant leftward shift in voltage‐dependent inactivation of atrial I_to_, with no significant shift in inactivation of ventricular I_to_.

### Effects of NS5806 on I_to_ restitution

**Figure 5:**
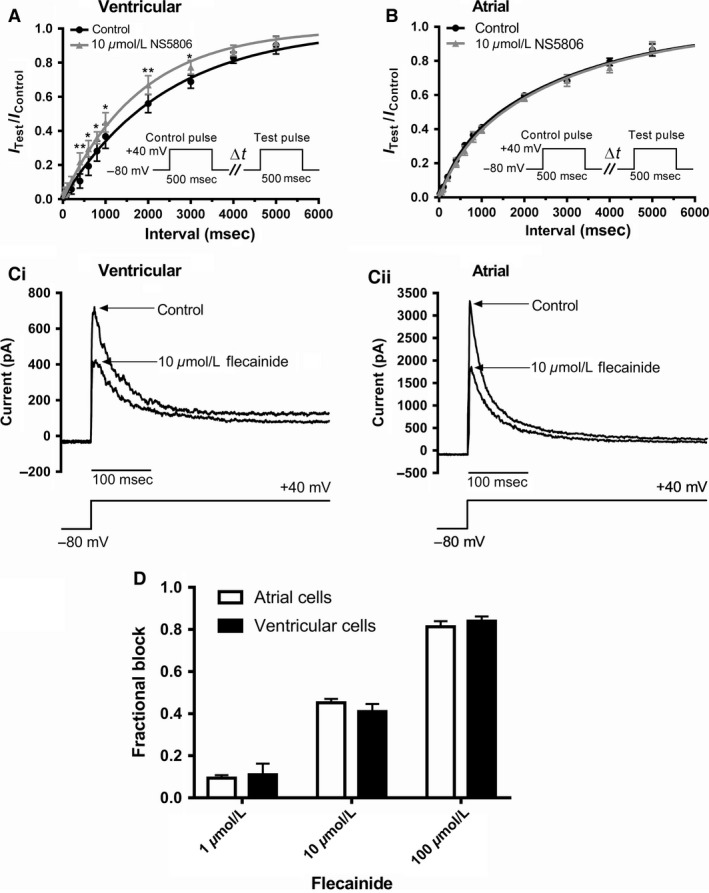
*Effect of NS5806 on recovery of I*_*to*_ *from inactivation (“restitution”) in atrial and ventricular myocytes and response to flecainide*. The protocol for studying restitution is illustrated in the insets to panels A and B: an initial 500 msec depolarizing step from −80 mV to +40 mV was followed by varying intervals (Δt, 20 msec to 5000 ms) at −80 mV followed by a ‘test’ depolarization to +40 mV. Each pair of pulses was separated by 10 sec. For each pulse‐pair, the magnitude of I_to_ elicited by the second command (I_T__est_) was expressed as the fraction of that elicited by the first (I_C__ontrol_). A: Mean (± SEM) data (*n* = 6) for recovery of I_to_ from inactivation for ventricular myocytes, in control (black) and 10 *μ*mol/L NS5806 (gray). Data were fitted by a single exponential function to get time constant values given in the Results. *difference between Control and NS5806 at *P* < 0.05; ** *P* < 0.01. B: Mean (± SEM) data (*n* = 7) for recovery of I_to_ from inactivation for atrial myocytes, in control (black) and 10 *μ*mol/L NS5806 (gray). Data were fitted by a bi‐exponential function to get time constant values given in the Results text. C: Representative traces of I_to_ in control and following exposure to 10 *μ*mol/L flecainide (same protocol as used in Figures 1 and 2) for ventricular (Ci) and atrial (Cii) myocytes. D: Bar chart plots for flecainide inhibition of ventricular and atrial I_to_ (*n* = 6–7 cells for each concentration for both cell types). 2‐way ANOVA with Bonferroni's post‐test confirmed that for each cell type the concentration dependence of the inhibitory effect was significant (*P* < 0.05), whilst at no concentration did the extent of inhibition differ significantly between atrial and ventricular cells.

In order to measure restitution of ventricular I_to_ (recovery from inactivation) a paired‐pulse protocol (shown schematically in the insets to Fig. 5A and B) was used (Mitcheson and Hancox 1999). Figure 5A shows mean data from six experiments in which restitution of I_to_ from ventricular cells was measured in control solution and following exposure to 10 *μ*mol/L NS5806. In both control and NS5806, I_to_ restitution followed a single exponential time course, with time constants of 2417 ± 117 msec and 1814 ± 82 msec in control and NS5806, respectively (*P* < 0.01, *n* = 6). Restitution of I_to_ from atrial cells (Fig. 5B) was best described by a bi‐exponential time course: the fast component had time constants of 452 ± 146 msec and 521 ± 287 msec in control and with NS5806, respectively, while for the slow component the corresponding values were 3023 ± 241 msec and 3045 ± 400 msec, respectively. The fraction of fast atrial I_to_ restitution was 21.2 ± 6.0% in control and 20.0 ± 10.8% in NS5806. None of these values differed significantly between control and NS5806 (*n* = 7). When restitution of atrial cell I_to_ was additionally fitted with monoexponential function to facilitate comparison with ventricular I_to_, this yielded time constants in control and NS5806, respectively of 2147 ± 57 msec and 2253 ± 71 msec (*n* = 7; *P* > 0.05). Taken together, these data indicate that NS5806 significantly accelerated restitution of I_to_ from rabbit ventricular cells, but did not significantly affect restitution of I_to_ from atrial cells.

### Effect of flecainide on ventricular and atrial I_to_

Since I_to_ arises from multiple channel isoforms the atrial‐ventricular differences in response to NS5806 might reflect different *functional* Kv1.4 and Kv4.x tissue expression. Flecainide has been reported to discriminate between Kv4.x and 1.4 channels, with the latter exhibiting lower sensitivity to inhibition by the drug (Yeola and Snyders 1997; Singarayar et al. 2003; Herrera et al. 2005). Effects of flecainide on rabbit ventricular and atrial I_to_ were therefore examined to probe the functional expression of these channel subunits. Figure 5Ci shows representative ventricular I_to_ traces in the absence and presence of 10 *μ*mol/L flecainide, whilst Figure 5Cii shows comparable data for atrial I_to_. The bar charts in Figure 5D show mean fractional block for I_to_ from the two cell types with 1, 10 and 100 *μ*mol/L flecainide (*n* ≥ 6 for each concentration). At no concentration did the inhibitory effect of flecainide differ significantly between atrial and ventricular cells. When the data for each cell type were fitted to standard concentration‐response relations to estimate IC_50_ values (constraining minimal and maximal possible fractional block values to 0 and 1, respectively; plot not shown) the derived value for ventricular I_to_ was 14.7 *μ*mol/L, (LogIC_50_ mean ± SEM = −4.83 ± 0.03, 95% C.I = 5.6–39.0 *μ*mol/L; n_H_ = 0.83 ± 0.05), whilst for atrial I_to_, the derived IC_50_ was 13.8 *μ*mol/L (LogIC_50_ mean ± SEM = −4.86 ± 0.05, 95% C.I = 3.2 to 60.3 *μ*mol/L; n_H_ of 0.79 ± 0.07). Thus, in contrast to their distinct responses to NS5806, ventricular and atrial I_to_ exhibited similar sensitivity to inhibition by flecainide.

### Effects of NS5806 on ventricular and atrial APs

**Figure 6:**
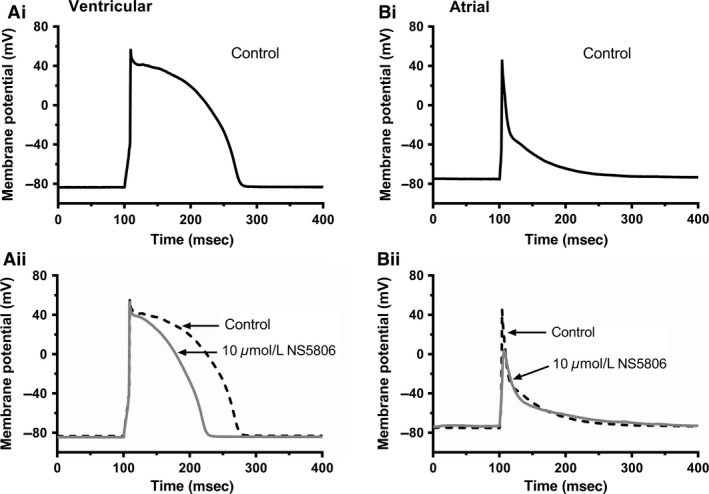
*Effect of 10 μ*mol/L *NS5806 on ventricular and atrial action potentials* Ai–Aii: Representative ventricular action potentials in control (Ai, black) and in 10 *μ*mol/L NS5806 (Aii, gray, with control action potential superimposed as dashed black line). Bi–Bii: Representative atrial action potentials in control (Bi, black) and in 10 *μ*mol/L NS5806 (Bii, gray, with control action potential superimposed as dashed black line). For A and B, depolarizing stimuli were applied at 2 sec intervals. Mean ventricular cell resting potential of −81.5 ± 0.7 mV was obtained with zero current injection. Atrial cell resting membrane potential was somewhat depolarized (~−50 to −40 mV) with zero current and so a small hyperpolarizing (−50 pA) current was injected to give the mean resting potential of −79.9 ± 1.9 mV. Mean action potentials parameters for both cell types in Control and NS5806 are given in Table 1.

In a final set of experiments, the action of 10 *μ*mol/L NS5806 on ventricular and atrial AP profiles was compared. For both cell types, APs were elicited in membrane potential (current clamp) recording mode, by brief (5–7 msec) duration suprathreshold depolarizing current pulses (0.6‐1 nA for ventricular myocytes and 0.4–0.5 nA for atrial myocytes) at a stimulation frequency of 0.5 Hz. Figure 6A shows representative ventricular APs in control and following application of 10 *μ*mol/L NS5806. The compound had no significant effect on the AP upstroke or initial overshoot (see Table 1); however, AP duration (APD) was abbreviated in the presence of the drug. We evaluated AP shortening at 20%, 50% and 90% repolarization (APD_20_, APD_50_, APD_90_), respectively. NS5806 shortened APD_20_ by 36.5 ± 5.0%, APD_50_ by 31.2 ± 3.3% and APD_90_ by 24.7 ± 3.0% (*n* = 7 for all; see Table 1 for absolute APD values). Figure 6B shows representative atrial APs in control solution and following application of 10 *μ*mol/L NS5806. In contrast to the AP shortening seen for ventricular APs, atrial APD was prolonged by the drug, particularly during early repolarization. APD_20,_ APD_50_ and APD_90_ were prolonged by 90.9 ± 14.7%, 88.6 ± 18.8% and 30.7 ± 12.0%, respectively (*n* = 7 for all; see Table 1). In addition, and in contrast to ventricular myocytes, atrial AP overshoot and upstroke were also affected (Table 1), with a marked (77.4 ± 3.8%) reduction in upstroke velocity in accord with dog atrial data in a previous report (Calloe et al. 2011). Further experiments with a higher concentration (100 *μ*mol/L) of NS5806 were not attempted, because the likely lack of selectivity of this concentration for ventricular I_to_ (Fig. 1C) would have make its effects on APs difficult to interpret.

**Table 1:** Effect of 10 *μ*mol/L NS5806 on action potentials from rabbit ventricular and atrial myocytes

	Ventricular cells (*n* = 7)		Atrial cells (*n* = 7)	
	Control	10 *μ*mol/L NS5806	Control	10 *μ*mol/L NS5806
Overshoot (mV)	56.0 ± 2.0	54.8 ± 2.0	43.5 ± 3.2	−2.3 ± 4.4 b
AP amplitude (mV)	137.5 ± 1.9	137.0 ± 2.0	123.2 ± 2.8	73.9 ± 3.8 b
Upstroke velocity (mV/msec) (percentage change,%)	128.4 ± 8.6	134.3 ± 7.0	120.9 ± 8.3	27.4 ± 4.9 b(−77.4 ± 3.8)
APD_20_ (msec) (percentage change,%)	73.6 ± 8.0	46.0 ± 4.9 b (−36.5 ± 5.0)	8.2 ± 1.1	16.0 ± 2.8 b(90.9 ± 14.7)
APD_50_ (msec) (percentage change,%)	133.8 ± 8.4	91.6 ± 6.4 b (−31.2 ± 3.3)	18.0 ± 2.8	32.9 ± 5.8 b(88.6 ± 18.8)
APD_90_ (msec) (percentage change,%)	167.4 ± 9.7	125.9 ± 8.8 b (−24.7 ± 3.0)	101.8 ± 7.3	128.6 ± 5.9 a(30.7 ± 12.0)

a *P* < 0.05.

b *P* < 0.01, paired *t*‐test.

## Discussion

### Comparison with prior canine ventricular and atrial I_to_ data

To our knowledge, this is the first study to investigate the concentration‐dependent effects of NS5806 on native cardiac I_to_. Previous work in dogs has shown that NS5806 increases the depth of phase 1 repolarization in both left and right ventricles in a concentration‐dependent fashion between 5 and 15 *μ*mol/L and, when phase 1 repolarization became very pronounced, could lead to AP collapse (Calloe et al. 2009). A lack of concentration‐response data on canine ventricular I_to_ for NS5806 means that direct comparison with our data is limited to the typical 10 *μ*mol/L concentration used in most prior dog studies (Calloe et al. 2010, 2011; Cordeiro et al. 2012). Table 2 compares the effects of NS5806 on rabbit and normal canine ventricular I_to_. The agonist effect of NS5806 at 10 *μ*mol/L is similar between the two species. However, concentration‐response data are not available for canine ventricular I_to_ to determine whether or not the biphasic concentration response relation we obtained at steady‐state is shared by the two species. Voltage‐dependent activation data are also lacking for dog I_to_, precluding comparison with the marked leftward shift in activation V_0.5_ found here for rabbit I_to_. Differences between the dog and rabbit I_to_ response to NS5806 are: (1) an apparent acceleration, not slowing of rabbit ventricular I_to_ inactivation time course with the compound (either as a result of direct inactivation modulation or some modest open channel block during the inactivating phase of the current); (2) no significant shift in voltage‐dependent inactivation V_0.5_ with NS5806 was seen in rabbit.

**Table 2:** Comparison of effects of NS5806 on normal rabbit and dog ventricular I_to_

I_to_ property	Rabbit	Source	Dog	Reference
*Ventricle*				
Current amplitude	Initial: ↑ EC_50_ 1.6 *μ*mol/L Steady state: “bell‐shaped” EC_50_ 1.6 *μ*mol/L; IC_50_ 40 *μ*mol/L	This study	↑ at 10 *μ*mol/L ↑ at 10 *μ*mol/L (Epi by 80%, Mid by 82% Endo by 16%)	Calloe et al. 2009 Calloe et al. 2010, 2011
Voltage dependence of activation	Negative shift in V_0.5_ of ~ −29 mV	This study	No data	
Time course of inactivation	Accelerated: t_half_ at +40 mV decreased from 30.3 msec to 21.5 msec (by 29%)	This study	Slowed: Tau at +40 mV increased from 12.6 to 20.3 ms (by 61%) I_to_ integral increased to 227%, 192% and 83% of control in EPI, MID and EPI	Calloe et al. 2009 Calloe et al. 2010;
Voltage dependence of inactivation	No statistical difference	This study	Negative shift in V_0.5_ of −6 mV EPI, −5 mV MID, −3.4 ENDO	Calloe et al. 2010 Calloe et al. 2011
Restitution	Accelerated: tau from 2417 msec to 1814 msec (by ~25%)	This study	Accelerated EPI and MID and biexponential to single exponential time course	Calloe et al. 2009 Calloe et al. 2010

The effects of NS5806 on rabbit atrial I_to_ differed significantly both from those seen in rabbit ventricular myocytes in this study and in canine atrial cells (summarized in Table 3). We observed a concentration‐dependent *inhibition* of atrial I_to_ amplitude (Fig. 2), accompanied by a ~−11 mV shift in voltage‐dependent activation (Fig. 3), a ~−3 mV shift in voltage‐dependent inactivation (Fig. 4), slowed inactivation time course, but unchanged restitution (Fig. 5). Canine atrial I_to_ was modestly increased (25%) by NS5806, and its restitution was accelerated – effects that differ markedly from those seen here in rabbit. No canine data are available on effects on voltage dependence of atrial I_to_ activation, whilst effects on I_to_ inactivation time course and voltage dependence are similar between the two species. The marked inhibitory effect of NS5806 on I_to_ accounts for atrial AP prolongation seen in our experiments (Fig. 6, Table 1). 10 *μ*mol/L NS5806 was reported to not alter phase 1 repolarisation in perfused dog atrial preparations, but shortened the APD_90_ (Calloe et al. 2011).

**Table 3:** Comparison of effects of NS5806 on normal rabbit and dog atrial I_to_

I_to_ property	Rabbit	Source	Dog	Reference
*Atrium*				
Current amplitude	↓ IC_50_ 18.2 *μ*mol/L	This study	↑ at 10 *μ*mol/L (25%)	Calloe et al. 2011
Voltage dependence of activation	Negative shift in V_0.5_ of ~ 11 mV	This study	No data	
Time course of inactivation	Slowed: t_half_ at +40 mV increased from 13.5 msec to 17.3 msec (by 28%)	This study	Slowed: Tau at +50 mV increased from 20 to 26.5 ms (by 32.5%)	Calloe et al. 2011
Voltage dependence of inactivation	Negative shift in V_0.5_ of ~ ‐3.3 mV	This study	Negative shift in V_0.5_ of ‐7.3 mV	Calloe et al. 2011
Restitution	No significant change	This study	Accelerated and biexponential changed to single exponential time course	Calloe et al. 2011

In atrial, but not ventricular myocytes, NS5806 produced a substantial slowing of AP upstroke velocity and amplitude (Fig. 6, Table 1), consistent with a selective reduction in atrial I_Na_. Such ‘off target’ actions of NS5806 on AP upstroke velocity were noted in dog atrial tissue and found to correlate with intrinsic atrial‐ventricular differences in I_Na_ inactivation kinetics that may favor atrial I_Na_ inhibition by the compound (Calloe et al. 2011). Thus, our own observations in respect of effects of NS5806 on atrial AP upstroke velocity are consistent with previously reported atrial‐ventricular differences in I_Na_ and atrio‐selectivity of drug I_Na_ modulation (Burashnikov et al. 2007; Calloe et al. 2011; Suzuki et al. 2013).

### On the mechanism of NS5806 action

The decrease in the slope factor for voltage‐dependent activation of ventricular I_to_ suggests that NS5806 either effectively alters the membrane field sensed by the I_to_ voltage sensor or decreases the net effective charge of the voltage sensor. The positive residues in the S4 region play a key role in forming the voltage sensor of Kv channels (for review see Swartz 2004), and since NS5806 should be negatively charged at pH7.2, it could decrease the slope of the activation curve by binding near the voltage sensor. However, NS5806 may also bind and exert effects outside the immediate S4 region. Consistent with this, NS5806 has been reported to produce an agonist action on Kv4.3/KChIP2/DPP6 channels expressed in mammalian CHO‐K1 cells and a smaller agonist effect on Kv4.3/DPP6 in *Xenopus* oocytes, whilst peak current carried by Kv4.3 alone was reduced by NS5806 (Lundby et al. 2010). The effects of NS5806 on inactivation (and recovery from inactivation) of Kv4.3 also seem to be sensitive to the interaction of NS5806 with KChIP2 (Lundby et al. 2010). Moreover, in canine ventricular myocytes, variation in response to NS5806 across the ventricular wall correlated with varying transmural KChIP2 expression levels in the presence of similar transmural levels of Kv4.3 (Calloe et al. 2010). Thus, to stimulate I_to_, it seems likely that NS5806 either interacts directly with the Kv4.3‐KChIP2 accessory subunit complex, or the interaction between Kv4.3 and KChIP2 exposes an interaction site for NS5806 on the Kv4.3 protein. In this regard, it is notable that a recent study investigating effects of NS5806 on the interaction between Kv4.3 and the KChIP2 relative KChIP3 has provided evidence that NS5806 binds at a hydrophobic site on the C terminus of KChIP3 and increases the affinity between KChIP3 and the N terminus of Kv4.3 (Gonzalez et al. 2014). Significantly, alignment of KChIP3 and KChiP2 (Uniprot Q9Y2W7 and Q9NS61, respectively) indicates that hydrophobic amino acid residues in KChiP3 (Tyr‐174 and Phe‐218) identified to be important for NS5806 binding (Gonzalez et al. 2014) are present in analogous positions in KChIP2, making it likely that the two interact similarly with NS5806.

Our data on ventricular I_to_ showed a biphasic concentration response relation to NS5806, with higher concentrations producing an initial stimulation followed by inhibition. In prior investigation of recombinant Kv channels, the response of Kv4.3/KChIP2/DPP6 to 100 *μ*mol/L NS5806 was smaller than that at 10 *μ*mol/L (see Fig. 2B in Lundby et al. 2010 at 100 *μ*mol/L –although this data‐point was excluded from the concentration‐response fit). In the same study, for Kv4.3/KChIP2 and Kv4.3/KChIP2/DPP6, concentrations up to 10 *μ*mol/L increased current amplitude and 30 *μ*mol/L produced some reduction (Fig. 3 in Lundby et al. 2010). In a different study directed toward the molecular pharmacology of hippocampal A‐current (based on Kv4.2 rather than Kv4.3), NS5806 increased Kv4.2/KChIP2 current amplitude at concentrations up to 20–60 *μ*mol/L, with an EC_50_ of 5.6 *μ*mol/L, but was inhibited at 200 *μ*mol/L (Witzel et al. 2012). Importantly, when Kv4.2/DPP6S or Kv4.2/KChiP3/DPP6a were co‐expressed, NS5806 produced a low‐affinity monophasic inhibition of the A current. These results support the idea that NS5806 interacts at more than one site to affect Kv4.x channels, with a lower affinity site, possibly on accessory subunits, mediating the inhibitory action. However, as Kv1.4 is inhibited by NS5806 (Lundby et al. 2010), an additional factor to be considered is contribution of Kv1.4 to the overall macroscopic rabbit I_to_. As shown in Fig. 5 (and discussed in more detail below), the similar sensitivity of ventricular and atrial I_to_ to flecainide argue against the differential effect of NS5806 on atria and ventricles being solely due to the presence of Kv1.4 in atria. Instead, it seems more likely that stimulation and inhibition combine, so that NS5806 acts as both an agonist and antagonist for ventricular I_to_ on the same channel complex(es). An additional unexpected feature of the response of ventricular cells to 100 *μ*mol/L NS5806 was the induction of a time‐dependent increase in outward current following initial inactivation of I_to_ (Fig. 1C). In principle, this could result from: (1) induction of an additional low NS5806 affinity gating mode of I_to_ or (2) some other off target effect (such as effects on the membrane or another current). The overall profile of the current in 100 *μ*mol/L NS5806 makes (1) unlikely; it seems improbable that I_to_ would inactivate then reactivate slowly during a test pulse to a fixed voltage. Off target membrane effects also seem less likely because 100 *μ*mol/L NS5806 did not produce a similar slow outward current in atrial cells (Fig. 2C). In addition, we tested for membrane effects in a limited number of additional experiments with a structurally closely related compound NS11021 (N′‐[3,5‐Bis(trifluoromethyl)phenyl]‐N‐[4‐bromo‐2‐(2H‐tetrazol‐5‐yl)phenyl]‐thiourea), which would be expected to have similar interactions with the cell membrane to NS5806. At 100 *μ*mol/L this compound did not produce a comparable slowly activating current to that with NS5806 in ventricular cells. Thus, it seems most likely that 100 *μ*mol/L NS5086 both affected ventricular I_to_ with biphasic time dependence (an increase followed by subsequent decrease in amplitude) and had an additional nonselective effect of activating another (unidentified) current. This secondary effect mitigates against the use of high concentrations of NS5806 for the selective enhancement of ventricular I_to_.

Our data on ventricular I_to_ inactivation and its modification by NS5806 have some notable similarities to those reported for Kv4.3 + KChIP2 expression in CHO cells by Calloe et al. (Calloe et al. 2010) in terms of V_0.5_ and *k*. However, the recovery from inactivation was slowed by NS5806 in that expression system unlike the acceleration seen both here and in dog (Calloe et al. 2009, 2010). This difference might be explained by heteromultimeric channel assembly (Po et al. 1993; Wang et al. 1999) which is encountered in many Kv channel families (for review see (Birnbaum et al. 2004)). In connection with this, heterologous expression produced by adding Kv1.4 subunits to an amphibian Kv4.3 expression system resulted in NS5806 speeding the recovery from inactivation (Lundby et al. 2010). However, in that expression system NS5806 had little effect on I_to_ amplitude which makes any simple translation of those results to the behavior of mammalian native cardiac I_to_ problematic.

The previously reported inhibitory effect of NS5806 on Kv1.4 (Lundby et al. 2010) together with our atrial I_to_ data might suggest a dominant role for Kv1.4 in rabbit atrial I_to_ as Kv4.3, 4.2 and 1.4 are all expressed in atria (e.g., Rose et al. 2005; Abd Allah et al. 2012)). However, antisense oligodeoxynucleotide probes show a slightly larger effect when directed against Kv4.3 than Kv4.2 and 1.4 (Wang et al. 1999; Bosch et al. 2003; Rose et al. 2005), so one would not expect a purely inhibitory effect of NS5806 in atria. In some preliminary experiments (not shown), 3 *μ*mol/L CP‐339,818, a compound which exerts preferential inhibition of Kv1.4 over 4.2 channels (Nguyen et al. 1996), partially inhibited both atrial and ventricular I_to_. Furthermore, the similar inhibitory potency of flecainide (as a probe to differentiate between Kv1.4 and Kv4.x channels) on ventricular and atrial I_to_ (Fig. 5) is not consistent with a more dominant role for Kv1.4 in atria as a lower atrial potency (compared to ventricle) should then occur (Yeola and Snyders 1997; Singarayar et al. 2003; Herrera et al. 2005) and this was not seen. An alternative explanation for the monophasic inhibitory effect of NS5806 on atrial I_to_ would be that KChIP2 has a weaker association with the Kv4.3 isoform in atria, a possibility that could be tested by examining the effect of NS5806 in KChIP2 native knockdown/overexpression systems in a future study.

Although rabbit ventricular I_to_ restitution time course is slower than reported for dog (Akar et al. 2004; Jost et al. 2013), the accelerated restitution of rabbit ventricular I_to_ produced by NS5806 (Fig. 5) is qualitatively similar to that seen in prior canine studies (Calloe et al. 2009, 2010). In contrast, the compound did not affect rabbit atrial restitution. These results differ markedly from the slowing of restitution by NS5806 seen for recombinant Kv4.3, even when KChIP2 is co‐expressed (Calloe et al. 2010; Lundby et al. 2010). This difference underscores present uncertainty as to the precise molecular makeup of native I_to_, and that caution is needed in the extrapolation of data obtained in expression systems to actual tissues.

### Functional relevance?

In our ventricular AP experiments, APs showed rapid initial repolarisation, but lacked an inscribed notch. Rabbit ventricular APs lacking a pronounced notch have also been seen in other studies (e.g., Giles and Imaizumi 1988; Kelly et al. 2013; Meedech et al. 2015). In our experiments, 10 *μ*mol/L NS5806 produced significant AP shortening at APD_20_‐APD_90_, an effect distinct from phase 1 repolarization (Fig. 6, Table 1). Incorporation of baseline rabbit I_to_ kinetics and of the I_to_ effects of NS5806 into a human ventricular AP model (O'Hara et al. 2011) qualitatively reproduced the experimentally observed AP shortening reported here (data not shown). In some respects, the ability of NS5806 to increase phase 1 and shorten phase 2 would oppose some of the deleterious changes seen in APs from failing human hearts. The restoration of the phase 1 notch in human should increase Ca^2+^ release synchrony (Cooper et al. 2010), whilst shortening of the AP should also reduce the duration of the Ca^2+^ transient (Cannell et al. 1987), via suppression of late release events (Cooper et al. 2010) as well stimulation of Ca^2+^ extrusion via sodium‐calcium exchange (Crespo et al. 1990). Consistent with this notion, recent data have shown that a dual I_to_ and I_Kr_ activator, NS3623, restores both the AP notch and protects against early after‐depolarisations in ventricular myocytes with reduced repolarisation reserve (Calloe et al. 2016).

## Conclusions

This study has demonstrated a biphasic concentration‐dependent modulation of rabbit ventricular I_to_ by NS5806 and a monophasic inhibitory effect of the compound on atrial I_to_. As both prior canine data and the present rabbit study indicate that NS5806 acts as a ventricular I_to_ agonist at the lower end of the *μ*mol/L range, it seems likely that at such concentrations the compound would also stimulate human native ventricular I_to._ However, at the same concentration as used in prior canine studies (Calloe et al. 2009, 2010, 2011; Cordeiro et al. 2012), NS5806 produced unexpected opposite effects on rabbit ventricular and atrial APD. Our ventricular data indicate that the consequences of I_to_ stimulation on ventricular repolarization can vary between species, depending on underlying I_to_ kinetics. The discordance between our rabbit atrial I_to_ data and prior canine atrial I_to_ data complicates extrapolation of these results to human atrial I_to_. With that *caveat*, whilst ventricular I_to_ activation might be anticipated to be beneficial in heart failure, concomitant atrial I_to_ inhibition could in principle promote initiation of re‐entrant arrhythmia in healthy atrial tissue if it promoted dispersion of atrial APD (Aslanidi and Hancox 2015). On the other hand, in a setting of electrically remodeled atria the APD lengthening effect of NS5806 could be beneficial and protect against sustained re‐entry (Aslanidi and Hancox 2015). Our data support the previously proposed notion that NS5806 additionally exerts atrio‐selective Na^+^ channel inhibitory effects (Calloe et al. 2011) and effects of combined atrial I_to_ and I_Na_ inhibition may well differ from those of I_to_ inhibition alone. Concomitant atrial I_Na_ inhibition by a ventricular I_to_ agonist may not be desirable unless abnormal atrial excitability is also present, and should be considered carefully during future design/development of such agents. Finally, the uncertainty as to the precise composition of native I_to_ channels means that the underlying basis of action of NS5806 and related molecules may best be further elucidated by the study of native rather than recombinant I_to_, combined with genetic modification of Kv and KChiP isoform expression.

## Conflicts of Interest

None.
